# MALDI-TOF MS for identification of Afro-tropical secondary malaria vectors

**DOI:** 10.1186/s12936-025-05549-6

**Published:** 2025-09-02

**Authors:** Mercy Tuwei, Jonathan Karisa, Caroline Wanjiku, Caroline Kiuru, Zedekiah Ondieki, Tobias Odongo, Festus Mure, Bruno Otieno, Peter Meli, Miguel Okoko, Brian Bartilol, Rehema Gona, Luis Constantino, Gildo Cole, Trisa Anastácio, Romário Armazia, Claudia Alves, Picardo Rui, Edith Ramaita, Martin Rono, Baltazar Candrinho, Joseph Mwangangi, Charles Mbogo, Derek Charlwood, Francisco Saute, Regina Rabinovich, Carlos Chaccour, Marta Ferreira Maia

**Affiliations:** 1 Kenya Medical Research Institute, Wellcome Trust Research Program, P.O. Box 230-80108, Kilifi, Kenya; 2 KEMRI Centre for Geographic Medicine Research - Coast, Kilifi, Kenya; 3 Division of National Malaria Programme, Ministry of Health, Nairobi, Kenya; 4 Centro de Investigação em Saúde de, Manhiça, Moçambique; 5 Instituto de Higiene e Medicine Tropical, Lisbon, Portugal; 6 Barcelona Institute for Global Health, Hospital Clínic – Universitat de Barcelona, Carrer Roselló 132, 08036 Barcelona, Spain; 7 CIBER de Enfermedades Infecciosas, Madrid, Spain; 8 Navarra Center for International Development, Universidad de Navarra, Pamplona, Spain; 9 National Malaria Control Program, Maputo, Mozambique; 10 Centre for Global Health and Tropical Medicine, University of Oxford, Oxford, UK

**Keywords:** Secondary malaria vectors, MALDI-TOF MS, *Anopheles cf. coustani 2 NFL-2015*, *Anopheles coustani*, *Anopheles rufipes*, *Anopheles pharoensis*, Species identification, Bio-typing, Mozambique, Kenya

## Abstract

**Background:**

Characterizing malaria epidemiology at the local level requires understanding the diverse malaria vector species driving transmission, including both primary and secondary vectors. Effective mosquito surveillance and accurate species identification are critical; however, due to the associated cost and complexity, most surveillance strategies mainly focus on the primary malaria vectors. There is a need for cost-effective methods that can reliably identify both primary and secondary vectors as their role in transmission becomes increasingly important while reaching towards elimination. This study aimed to evaluate the use of MALDI-TOF MS as a sustainable tool for identifying secondary malaria vector.

**Methods:**

Adult mosquitoes were collected in Kenya and Mozambique and morphologically identified. Secondary malaria vectors were considered as any Anopheline that did not pertain to *Anopheles gambiae *sensu lato (*s.l*.). or *Anopheles funestus *sensu lato (*s.l.).* At KEMRI Wellcome Trust Research Programme, MALDI TOF MS spectra were obtained from individual cephalothoraxes. Library creation and querying were guided by confirmatory species identification using Sanger sequencing of a subset of mosquitoes, targeting the Internal Transcribed Spacer 2 (ITS2) region of nuclear ribosomal DNA and the mitochondrial Cytochrome c Oxidase Subunit I (COI) gene. The libraries were then applied for the identification of other secondary malaria vectors.

**Results:**

Species identification of secondary malaria vectors using MALDI-TOF MS showed high concordance with Sanger sequencing with an overall accuracy of 91% and a kappa value of 0.87. The technique demonstrated a sensitivity and specificity of 100% for most species, except for distinguishing between *Anopheles cf. coustani 2 NFL-2015* and *Anopheles ziemanni*. In Kenya, the *Anopheles* species identified were *Anopheles cf. coustani 2 NFL-2015* (19), *Anopheles pretoriensis* (6), *Anopheles rufipes* (8), *Anopheles ziemanni* (8), *Anopheles coustani* (2), and *Anopheles pharoensis* (1). In Mozambique, the identified species comprised: *An. cf. coustani 2 NFL-2015* (10), *An. pretoriensis* (2), *An. ziemanni* (7), *An. coustani* (28), and *An. pharoensis* (4).

**Conclusion:**

The results provide evidence that MALDI-TOF can identify secondary malaria vectors from Eastern and Southeastern African regions. This technique was as efficient as DNA sequencing in identifying mosquito species. Indeed, except for *An. cf coustani 2NFL-2015* and *An. ziemanni*, an exact species identification was obtained for all individual mosquitoes. These findings highlight the potential of MALDI-TOF MS for monitoring malaria vectors.

## Background

Despite ongoing efforts to control and eliminate malaria, progress has stalled since 2015 [1]. This stagnation is attributed to insecticide and parasite resistance [2], behavioural changes in primary vectors [3], and the emergence of secondary and novel vector species. Secondary vectors contribute significantly to residual transmission due to their distinct biting and resting behaviours, which often render them less susceptible to current interventions such as insecticide-treated nets (ITNs) [4] and indoor residual spraying (IRS) [5, 6].

These vectors have been found to be infected with *Plasmodium* parasites, and in some settings, may play a significant role in transmission [7, 8]. Several Anophelines have been identified as secondary malaria vectors, including *Anopheles squamosus, An. tenebrosus*[9, 10], *An. coustani*, *An. ziemanni*, and *An. rufipes* [11]. Despite playing a potential role in sustaining malaria transmission, given their large numbers [12], secondary malaria vectors have been largely overlooked. There is insufficient data on their distribution, behaviour, and vector competence–preventing informed decision-making targeting these vectors. Improved understanding of their role in malaria transmission in different settings is needed to reach elimination. However, control and research programmes often lump these vectors as “other anophelines” and go no further in trying to identify them to species. Morphological identification is difficult with many sharing similar taxonomical features [13]. Furthermore, members of species complexes like *An. coustani *sensu lato (*s.l.*) are morphological identical but exhibit different behavioural traits requiring molecular methods to tell them apart.

DNA-based methods can be used to precisely identify species. However, these methods are resource-intensive and given that secondary vectors are not often a priority for control programs, they are rarely performed. The need for more cost-effective and scalable tools has led to the exploration of alternative technologies for mosquito surveillance. Matrix-Assisted Laser Desorption Ionization-Time of Flight Mass Spectrometry (MALDI-TOF MS) has emerged as a promising solution [14]. This innovative technology, originally developed for microbial identification [14], works by analysing protein spectra from biological samples, offering a rapid, accurate, and high-throughput approach to species identification. In the context of mosquito surveillance, MALDI-TOF MS has a demonstrated ability to accurately identify sibling species of the primary malaria vector complexes (*An. gambiae s.l.* and *An. funestus s.l*.) [15]. Unlike traditional methods, MALDI-TOF MS combines speed and reliability, requiring minimal sample preparation and expertise once reference libraries are established. The adoption of MALDI-TOF MS in mosquito surveillance has the potential to transform our ability to monitor vector populations, particularly in areas with high species diversity and limited resources for molecular assays. This study aims to evaluate the utility of MALDI-TOF MS for the identification of secondary malaria vectors and comparing its accuracy to morphology and DNA-based molecular assays.

## Methods

### Study sites

Entomological collections were conducted in two countries: Kenya and Mozambique.

In Kenya, sampling was done in Kwale, Kilifi, and Taita-Taveta counties as previously described [7, 8]. In Taita-Taveta, collections were performed in two villages: Kimundia and Kitobo. Kimundia is characterized by large banana plantation while Kitobo borders Tanzania with small scale rice and banana farming. In both sites households kept goats, dogs, cats, chicken and cattle. In Kwale, mosquitoes were collected in the context of the BOHEMIA trial [16] in the coastal sub-counties of Lungalunga and Msambweni, Ramisi and Pongwe-Kikoneni wards. The site was characterised by large rice and sugar cane farms supported by irrigations schemes as well as subsistence farming. Cattle and goats were found in high numbers throughout the area. In Kilifi samples were collected in Garithe which is a relatively dry area characterized by shrubs and subsistence farming of mainly cassava and coconut [7, 8]. In Mozambique, mosquito collections were conducted as part of the BOHEMIA trial site in Mopeia, located in the Zambezia region. Mopeia lies along the Zambezi River, which frequently overflows onto the flood plains during the long rainy season. Cattle were scarce in the area, with pigs and ducks being the predominant livestock.

### Sample collection, and mosquito species identification

Adult host-seeking mosquitoes were sampled using CDC light traps (Mozambique, Mopeia and Kenya, all counties) and Furvela tent traps (Kenya, Taita-Taveta) [17, 18]. CDC light traps were deployed indoors near sleeping areas and outdoors near animal enclosures, operating between 17:00 and 07:00. Furvela tent traps were set approximately 5–10 m from a house, where two occupants slept inside. A modified light trap was attached to the tent opening to capture mosquitoes attracted to human odors as they approached the netting. Resting mosquitoes were collected (Mozambique, Mopeia and Kenya, Kwale) indoors using Prokopack aspirators and outdoors from the walls of dug out pit shelters. After sorting, mosquitoes were morphologically identified to genus and species complex level [19]. However, not all specimens could be morphologically identified due to their physical condition. Mosquitoes were classified as secondary malaria vectors if they did not belong to the main complexes: *An. gambiae s.l.* or *An. funestus s.l.* All samples were stored individually in silica gel and transported to KEMRI Wellcome Trust Research Programme (KWRTP) in Kilifi, Kenya, for further analysis.

### Sample processing

Mosquitoes were dissected into three anatomical parts: 1) cephalothorax; 2) legs and wings; and 3) abdomen. The cephalothoraxes were longitudinally severed into equal halves: one half was used to collect a MALDI-TOF spectra. The other half was used to confirm species the species ID using Sanger sequencing, and infection with *Plasmodium* spp. using PCR.

### Protein extraction

Mosquito cephalothoraxes (half) were homogenized in 15 µl of 70% (v/v) formic acid (Thermo scientific, Czech Republic) and 15 µl of 50% (v/v) acetonitrile (ACN) (Thermo scientific, USA) in 106 µm acid wash glass beads (Sigma-Aldrich, USA) using a TissueLyser II (Qiagen, Germany) machine (3 cycles of frequency 30 Hz for 1 min) followed by centrifugation at 15,000 rpm for 30 s [20]. Thereafter, 2 µl of the supernatant were spotted on MALDI-target (Bruker Daltonics) steel plate in quadruplicate [20]. The steel plate was left to air dry for a few minutes, after which, each sample target well was overlaid with 2 µl matrix solution (α-cyano-4-hydroxycynnamic acid (Sigma-Aldrich, USA) 50% (v/v) acetonitrile, 2.5% (v/v) trifluoroacetic acid (Thermo scientific, USA) and 47.5% LC–MS grade water (Thermo scientific, USA)). The steel plate was allowed to dry before loading onto a MALDI TOF MS Biotyper (Bruker Daltonics) machine for spectra acquisition.

### MALDI TOF MS spectra acquisition and analysis

Spectra were acquired using the FlexControl software ver. 3.3.0 (Bruker Daltonics) with a slight modification on the laser power of 50% while the rest of were at default setting [7]. To ensure the instrument is functioning optimally and generating high-quality spectra, we routinely use *Bruker Test Standard (BTS)* as a control. The resulting spectra were then exported to FlexAnalysis software ver. 3.3.0 (Bruker Daltonics) for spectra cleaning by checking their reproducibility, intensity and baseline smoothing, poor quality spectra and flat lines were removed. A sub-sample of the good quality spectra were randomly selected for species identification databases creation, while the remaining spectra were used for validation by querying the library database created. All good quality spectra were exported to MALDI Biotyper explorer software ver. 3.3.0 (Bruker Daltonics) for further spectra smoothing and normalization. Dendrograms were created from the main spectral profiles (MSPs) based on the species category.

### Library database creation and validation

MALDI TOF MS library database for the species identification were created using MSPs generated by MALDI Biotyper explorer software ver. 3.3.0 (Bruker Daltonics). For each species category, a minimum of 3–5 spectra were chosen for representation in the library and database creation. Spectra that had good reproducible and intensity were loaded onto MALDI Biotyper and further subjected to smoothing, checking for intensity, frequency, and peak picking position of the MSP spectra. Validation was performed on the remaining spectra by querying the unknown spectra of different mosquito species with the reference spectra in the database. The MALDI Biotyper calculates a log score value that ranges from 0 to 3, indicating the level of match between the unknown spectra and the reference database. An LSV ≥ 1.8 was considered as reliable identification.

### Molecular analysis

DNA was extracted from the remaining cephalothorax half using Chelex method [21]. In brief, 80 µl Chelex solution was added into each sample, the tissue lysed, heated for 10 min at 95 °C followed by centrifugation for 10 min. The supernatant (containing the DNA) for each sample was then transferred into a clean tube until further processing [7]. The DNA was immediately analysed or stored in − 20 °C, awaiting further processing.

The extracted DNA was amplified using primers targeting the ribosomal DNA internal transcribed spacer region 2 (ITS2). Samples that did not amplify in the ITS2 assay were amplified using primers targeting the cytochrome oxidase subunit 1 (*CO1*) loci of the mitochondrial DNA(13). A reaction PCR mix volume of 11 μl was composed of 5 μl of GoTaq, 1 μl of the reverse and forward primers, 5 μl of nuclease free water and 5 μl of DNA template. The thermocycler conditions for ITS2 were as follows: initial denaturation of 95 °C for 3 min, followed by 35 cycles of 95 °C for 30 s, 52 °C for 1 min, 72 °C for 30 s representing denaturation, annealing and elongation, respectively. The final elongation was 72 °C for 10 min. For COI, the annealing temperature was 53 for 1 min. To check for successful amplification, the resulting PCR products were visualised on 1.5% gel using Bio-Rad UV imager. All samples (amplicons) that were purified using Exo-SAP Enzyme purification kit. Briefly, 2 µL of ExoSAP was added to each well containing 5 µL of post-PCR product, followed by incubation at 37 °C for 4 min and 80 °C for 1 min. The purified DNA amplicons were subjected to bi-directional Sanger sequencing following the manufacturer’s instructions. BigDye sequencing was performed on all amplified samples using a PCR master mix (total volume of 8 µL), consisting of 0.5 µL BigDye enzyme, 1.75 µL buffer, 5.25 µL water, 1 µL forward and reverse primers, and 2 µL DNA template. The generated sequence chromatograms were checked for quality and indels (insertion and deletion) using Bio-edit (version 7.2.5, 2013) [22] contigs were generated. The contigs were searched against reference DNA nucleotide sequences database in the NCBI (National Center for Biotechnology Information) using Basic Local Alignment Search Tool (BLASTn). Sequences/contigs with a ≥ 98% sequence percent identity against the reference DNA nucleotide NCBI database were considered. However, contigs with a sequence percent identity ≤ 95% were considered inconclusive.

### Plasmodium detection

*Plasmodium* spp. screening was done by PCR using primers targeting the 18 s of the ribosomal DNA [23]. Successful amplification of the resulting PCR products was visualised on 1.5% gel using Bio-Rad UV imager.

## Results

### Mosquito collections

A total of 1,228 secondary malaria vector were collected across all sites. In Mozambique, 547 samples were analysed. Morphological identification indicated the presence of comprising *An. tenebrosus* and *An. coustani*. In Kenya, sampling was conducted in three locations: Kwale, Taveta and Kilifi. In Kwale, 158 samples were collected but were not morphologically identified and were therefore classified as “other *Anopheles*”. In Taveta, 521 samples were collected and morphologically identified as tentatively: *An. tenebrosus*, *An. coustani*, *Anopheles erepens*, and *Anopheles pharoensis*. In Kilifi, two *An. coustani* were morphologically identified.

### Selection of samples for sequencing

#### Sample selection and species identification using molecular methods

Following spectral acquisition, only high-quality spectra were selected for sequencing. A quality spectra is characterized by the presence of high-intensity, well-resolved peaks, reproducibility across replicates spectra, species-specific peak patterns, and a stable, flat baseline. Spectra selection was guided by morphological identification where available, as not all mosquitoes had been morphologically identified. A total of 176 samples with high-quality samples were successfully sequenced using Sanger sequencing. Sequencing results were used to inform and validate the MALDI-TOF MS library creation by careful characterisation of the reference spectra (MSPs) and confirmation of query results. From these, 95 were used for database creation, validation and quality control which were *An cf. coustani* 2 NFL-2015 (29), *An. coustani* (30), *An. pharoensis* (5), *An. rufipes* (8), *An. ziemanni* (15), and *An. pretoriensis* (8). The remaining 81 samples were excluded as they were identified as *An. funestus *sensu stricto (*s.s.*) (49), *Mansonia uniformis (24), Culex bitaeniorhynchus* (1), *Anopheles cf. rivulorum* (1), *An. rivulorum* (2), or poor-quality sequence (4).

### MALDI TOF analysis

#### Spectra acquisition, database creation

**Fig. 1:**
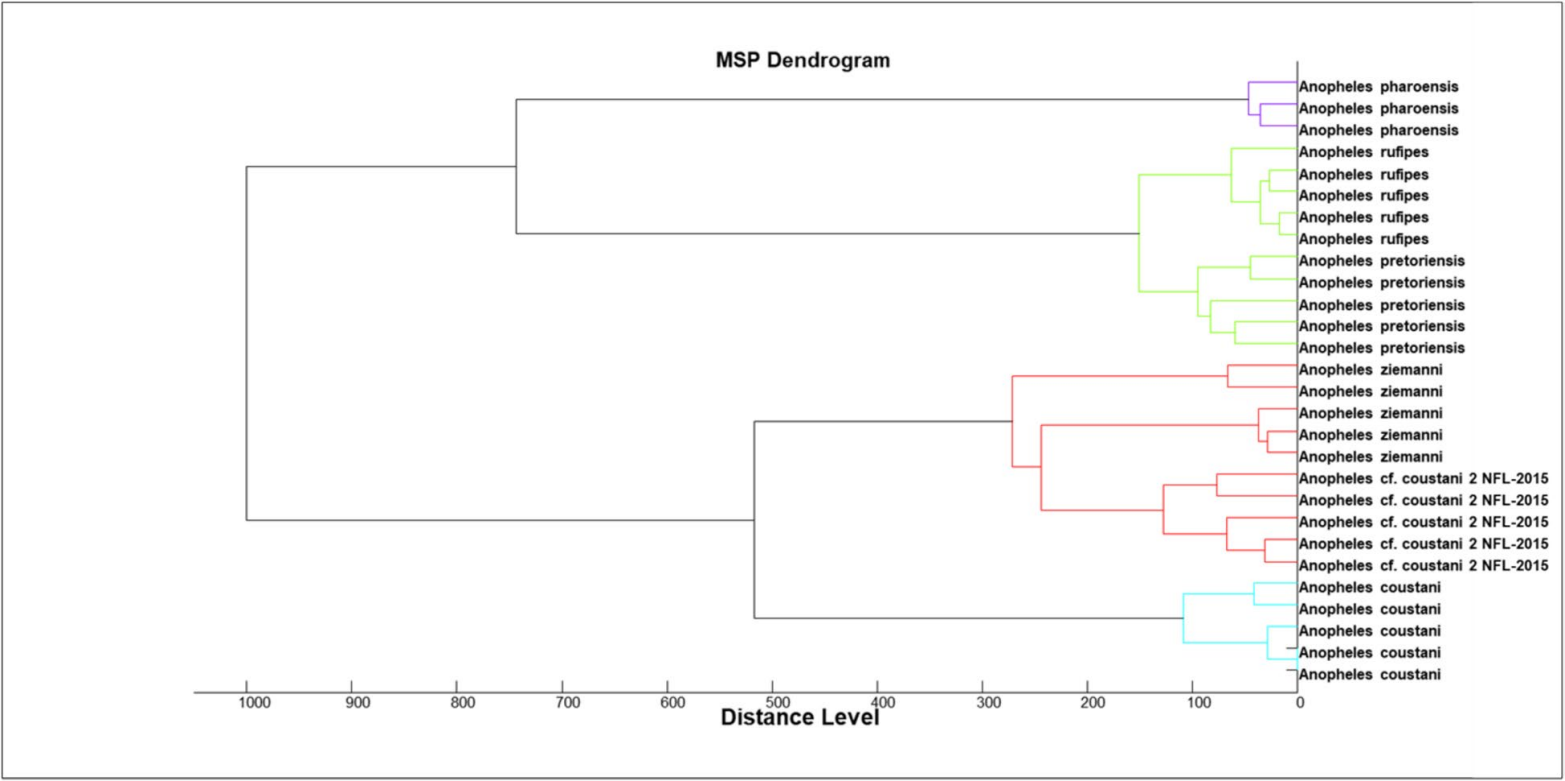
Dendrogram showing a clear separation between the different species used for database creation

Spectra were acquired from all the 1,228 samples. Among the 95 confirmed secondary malaria vectors, 27 (28%) were used for database creation, *An. cf. coustani 2 NFL-2015* (5), *An. coustani (5)*, *An. pretoriensis (5)*, and *An. rufipes (5), An. ziemanni* (4), and *An. pharoensis* (3). Spectra selected from each species were used to generate dendrogram and showing a distinct separation between *An. coustani*, *An. pretoriensis*, *An. rufipes, while coustani 2 NFL-2015* and *An. ziemanni* they were closely related (Fig. 1).

### Database validation and quality control

**Fig. 2:**
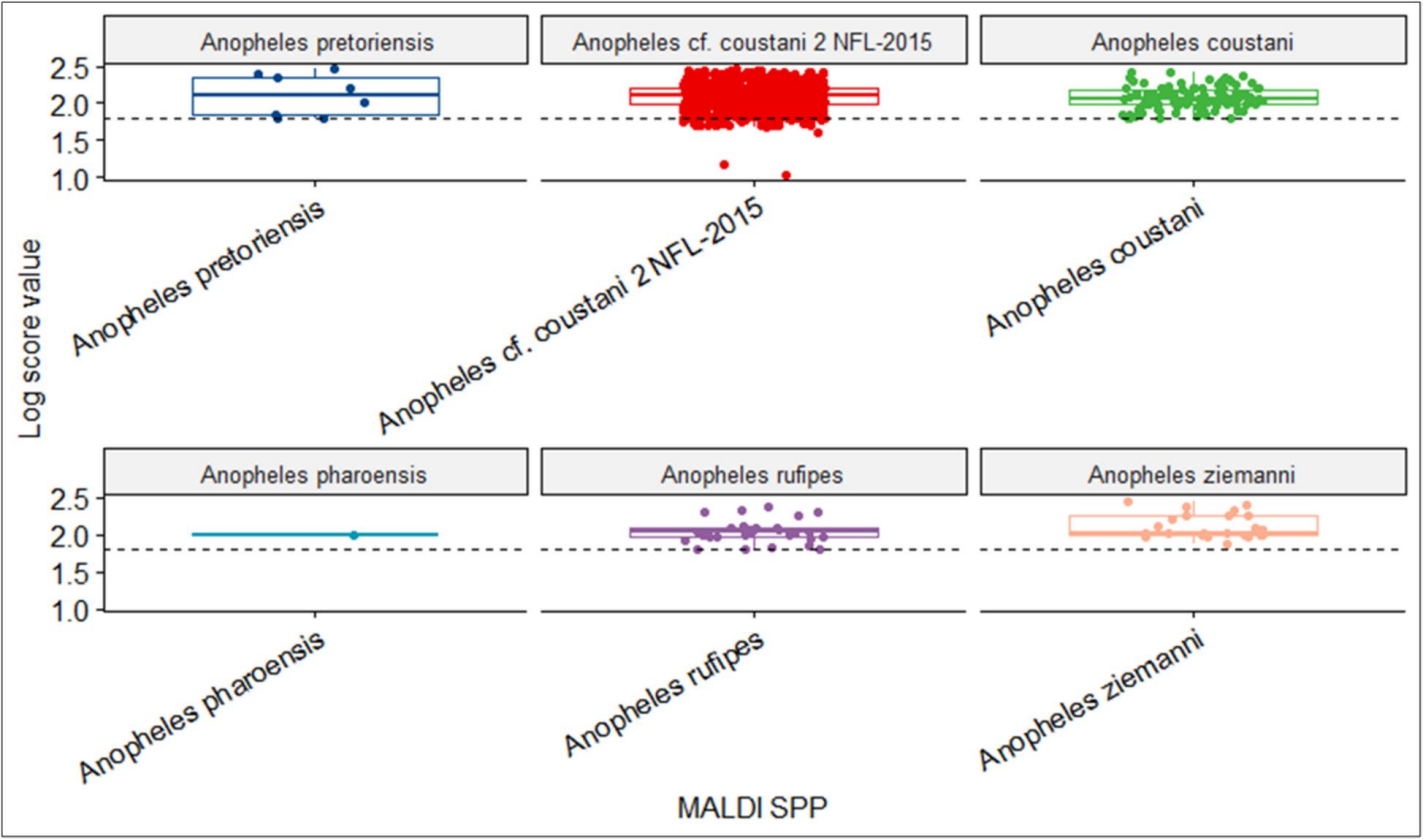
Box plot showing log score values of 1,052 samples queried with the library created

A total of 68 samples were designated for validation and quality control. Log score values exceeding 1.8, confirmed accurate identification (Fig. 2), yielding results with overall accuracy of 91% across all categories. Sensitivity and specificity for all the species was 100% apart from *An An. cf. coustani 2 NFL-2015* and *An. ziemanni* which had a sensitivity of *83% and* 82%, respectively. A Cohens’ Kappa of 0.87 was reported, signifying perfect agreement between MALDI-TOF MS predictions and sequencing observations (Table 1).

**Table 1:** Performance of MALDI­TOF MS in predicting secondary malaria vectors species

Sequencing						
Prediction MALDI TOF MS	*An. pretoriensis*	*An cf. coustani 2 NFL-2015*	*An. coustani*	*An. rufipes*	*An. pharoensis*	*An. ziemanni*
*An. pretoriensis*	3	0	0	0	0	0
*An cf. coustani 2 NFL-2015*	0	20	0	0	0	2
*An. coustani*	0	0	25	0	0	0
*An. rufipes*	0	0	0	3	0	0
*An. pharoensis*	0	0	0	0	2	0
*An. ziemanni*	0	4	0	0	0	9
Sensitivity	1	0.83	1	1	1	0.82
Specificity	1	0.95	1	1	1	0.93
Accuracy	0.91					
Cohens’ kappa	0.87					

### Library query

Following database validation with the sequenced samples (68), confirming 91% concordance with sequencing, 1,052 samples that had not been sequenced were queried using the MALDI-TOF MS library that was created. MALDI-TOF MS identified with certainty (LSV above 1.8): 747 as *An. cf. coustani 2 NFL-2015*, 73 as *An. coustani*, 5 as *An. pretoriensis*, 27 as *An. rufipes*, and 10 as *An. ziemanni*. There was considerable discordance with the morphological identification with some samples being confirmed as primary malaria vectors (148) by MALDI-TOF MS or even Culicines (*Mansonia uniformis* (33) (Table 2).

**Table 2:** MALDI TOF MS species prediction results of samples morphologically identified as secondary malaria vectors queried using the library here developed denoting poor accuracy of morphological identification

	*An. erepens*	*An. tenebrosus*	*An. coustani*	*An. pharoensis*	Other *Anopheles*	N
MALDI.SSP.ID:	N = 2	N = 463	N = 416	N = 55	N = 116	1052
*An. funestus s. s*	0	0	89	0	0	
*An. gambiae s. s*	0	2	16	0	0	
*An. quadriannulatus*	0	0	0	0	1	
*An. rivulorum*	0	0	0	0	16	
*An. arabiensis*	0	8	12	0	4	
*An. cf. coustani 2 NFL-2015*	0	360	282	41	64	
*An. coustani*	0	60	6	0	7	
*An. pretoriensis*	3	1	0	0	1	
*An. rufipes*	0	0	4	0	23	
*An. ziemanni*	0	0	2	8	0	
*Mansonia uniformis*	0	25	2	6	0	
Poor quality	0	6	3	0	0	

### Sporozoite detection.

All mosquitoes (n = 1,228) were analyzed by PCR assay to confirm the presence of *Plasmodium falciparum*. None of the secondary malaria vectors tested positive for parasite infection.

## Discussion

This study demonstrates that MALDI-TOF technology can accurately identify secondary malaria vector species, further confirming the potential role of MALDI-TOF MS for malaria vector surveillance in Africa and enhancing the available toolkit for vector surveillance. The reference libraries created in this study complement existing reference libraries previously developed for primary malaria vectors [20] which also achieved high prediction accuracy (97.5%) for *An. gambiae s.l*. and *An. funestus s.l*.

The prediction accuracy of MALDI TOF MS validated by Sanger sequencing, demonstrating 91% accuracy in distinguishing different species of secondary malaria vectors with a kappa value of 0.87, indicating excellent MALDI-TOF MS concordance with sequencing. Misclassifications were only reported between *An. cf. coustani 2* NFL-2015 and *An. ziemanni*. This may be attributed to their highly similar protein profiles, denoting their close genetic relatedness. Previous studies have reported that some members of the *An. coustani* group are difficult to distinguish even using molecular markers, further supporting this observation [24]*.* Furthermore, the library generated in this study only included five reference spectra per species. It is likely that with further addition of MSPs representing these two species the methods prediction accuracy will increase.

It was noted with concern that morphological identification of secondary vectors often resulted in misclassifications, with some cases these being primary Anopheline vectors, or even Culicines [25]. This concern underscores the urgent need for advanced tools like MALDI-TOF MS to enhance mosquito species identification and improve vector surveillance. Even though all teams received training in taxonomy and technicians are regarded as “experts,” relying solely on morphological data inevitably leads to errors, potentially misguiding control programs. Secondary malaria vectors are generally distinct from primary vectors; however, poor morphological identification can be especially problematic when species like *Anopheles stephensi* closely resemble *An. gambiae*, increasing the risk of misidentification. Although secondary vectors have been implicated in malaria transmission before, including in Taita-Taveta where some collections were conducted [8], no Plasmodium-infected secondary vector detected at any of the study sites.

Effective malaria vector surveillance requires a clear understanding of local vector composition and their role in transmission. Implementing a cost-efficient species identification tool can significantly enhance surveillance programs, especially during the elimination stage when all sources of transmission must be targeted. MALDI-TOF MS is already incorporated into the European Centre for Disease Prevention and Control (ECDC) guidelines for monitoring invasive mosquitoes [26] and is widely used for this purpose in several European countries [27, 28]. As a cost-effective, high-throughput technology, it can accurately differentiate closely related mosquito species, making it an asset for vector surveillance. Its effectiveness depends on well-developed spectral libraries, which, once established, enable precise species identification. For malaria-endemic regions, MALDI-TOF MS offers several practical advantages. It utilizes existing microbiology laboratory infrastructure, aligns with clinical diagnostics to encourage investment, and seamlessly integrates into laboratory workflows without requiring specialized training. Additionally, its consistent performance across different sites, supported by a single reference library, ensures reliable identification. The technology also benefits from intuitive, readily available analytical software, simplifying data interpretation and making it accessible to a wide range of users without the need for advanced data analysis skills. Although many laboratories in Africa already use MALDI-TOF MS for species identification [29], spectral libraries remain largely institution-specific. Following the MALDI-TOF MS network models in the microbiology field, a user network for spectra sharing should be established to maximize the impact of this technology in vector monitoring and surveillance and to encourage broader adoption.

## Conclusions

This study highlights the utility of MALDI-TOF MS for identifying secondary malaria vectors, demonstrating its strong concordance with molecular methods. By overcoming the limitations of morphological identification and the costs of sequencing, MALDI-TOF MS offers a reliable, cost-effective, and scalable tool for vector surveillance. Expanding reference libraries and establishing a shared spectral network will be critical for maximizing its impact across malaria-endemic regions, particularly in supporting elimination efforts through precise vector monitoring.

## Data Availability

The supporting data is under the custodianship of the KEMRI-Wellcome Trust Data Governance Committee and is accessible upon request addressed to that committee. [10.7910/DVN/GEYSM1] (https:/doi.org/10.7910/DVN/GEYSM1)).

## Abbreviations

ITS2: Internal transcribed spacer
COI: Cytochrome c oxidase subunit I
*s.l*.: sensu lato
DNA: Deoxyribonucleic acid
ITNs: Insecticide-treated nets
IRS: Indoor residual spraying
